# "It Looks Arterial": The Chest X-Ray of Nightmares

**DOI:** 10.7759/cureus.79532

**Published:** 2025-02-23

**Authors:** Tianyu Chen

**Affiliations:** 1 Hospital-Based Medicine, University of California Los Angeles, David Geffen School of Medicine, Los Angeles, USA

**Keywords:** central line misposition, dialysis catheter placement, hemiazygos, procedural complication, ultrasound

## Abstract

Central venous access is a common procedure for a variety of indications such as dialysis, plasmapheresis, or difficult venous access. Although a relatively safe procedure that can be done at the bedside, complications can still occur. Recognition and management of central line complications such as vascular injury and bleeding, infection, and misplacement are important when caring for patients with central vascular access and for the proceduralist who places the central line. Here we present an uncommon complication of internal jugular central access where a left internal jugular central line placement resulted in misplacement of the line in the hemiazygos vein.

## Introduction

Central venous access is a common procedure for a variety of indications such as dialysis, plasmapheresis, or difficult venous access. Although a relatively safe procedure that can be done at the bedside, complications can still occur. A central venous catheter can be inserted into a vein in three different sites bilaterally: the internal jugular, subclavian, or femoral veins [1]. The right internal jugular is the most direct path to the right atrium via the superior vena cava (SVC), where we want the central line catheter tip to lie, and thus have low rates of catheter misposition. The left internal jugular poses a more common complication of mispositioning given there is a longer and more oblique route to the SVC [2]. The femoral veins are compressible sites and may be more appropriate for patients who are at higher risk for bleeding. The subclavian approach has a higher risk for pneumothorax [3]. Recognition and management of central line complications such as vascular injury and bleeding, infection, and misplacement are important when caring for patients with vascular access and for the proceduralist who places the central line.

## Case presentation

**Figure 1:**
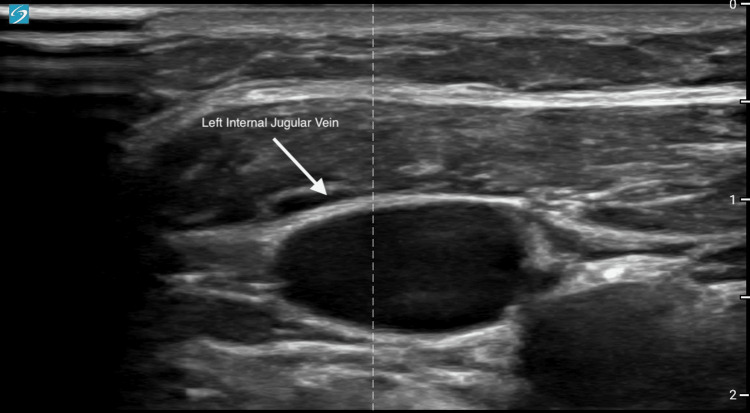
Cross-sectional ultrasound image of the left internal jugular vein

A 57-year-old female patient with a history of type 1 diabetes, hyperlipidemia, hypertension, and end-stage renal disease status post-kidney transplant presented for a temporary dialysis catheter placement for plasmapheresis due to transplant rejection. An evaluation of her internal jugular and femoral veins bilaterally was conducted with ultrasound. She had deep and small femoral veins bilaterally, a stenosed right internal jugular, and a favorable shallow and big left internal jugular vein (Figure 1). The decision was made to place the dialysis catheter in the left internal jugular vein.

**Figure 2:**
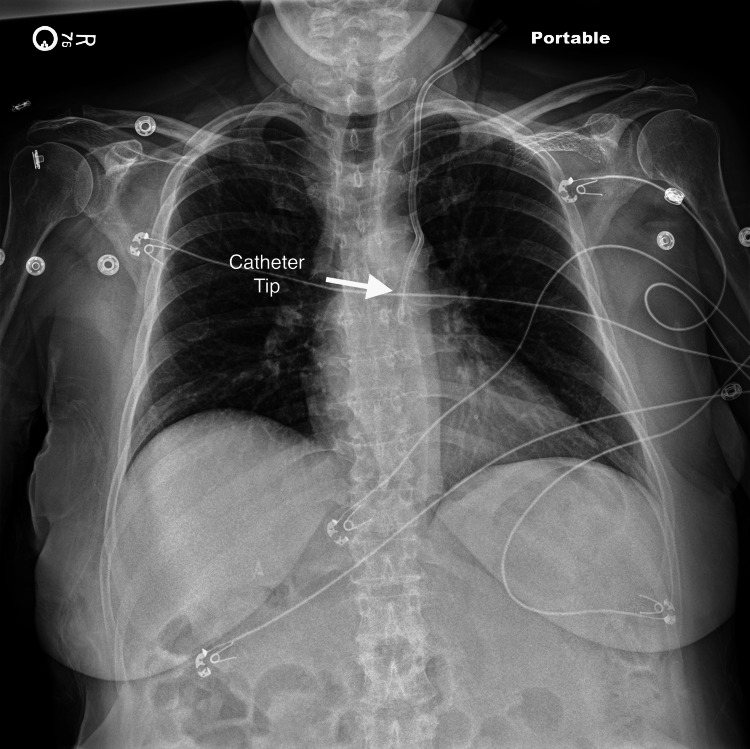
Chest X-ray post central line placement with catheter remaining on the left side of chest

**Figure 3:**
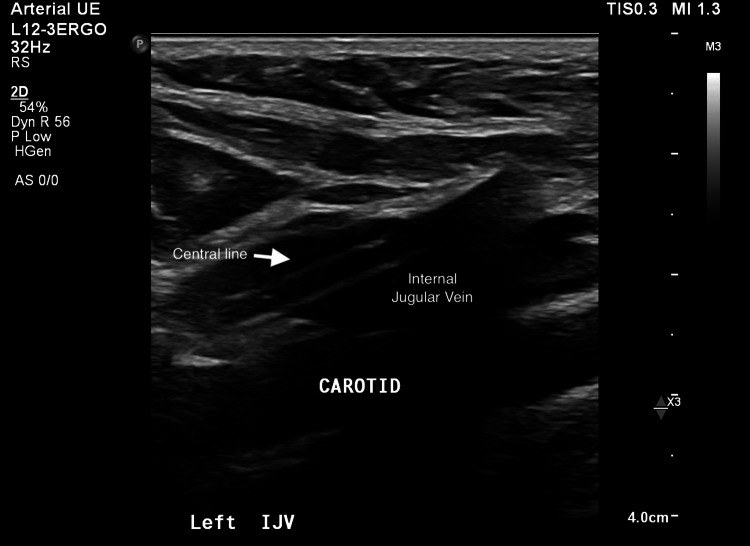
Ultrasound confirmation of central line positioning within the internal jugular vein with the carotid patent IJV: internal jugular vein (long axis view)

During catheter placement, all steps of the procedure with the Seldinger technique for venous access went smoothly and the patient remained hemodynamically stable throughout the procedure and afterward. The placement of the line went without any resistance and the position of the catheter in the left internal jugular vein was verified on ultrasound. However, a routine post-placement chest X-ray revealed that the catheter did not cross the midline and descend into the SVC (Figure 2). The proceduralist called radiology to get a stat read on the X-ray and the radiologist stated that the catheter looked like it was placed arterially, which can be a fatal complication of central line placement. The radiologist reviewed the patient’s previous CT chest and noted a very large hemiazygos vein. Although it could not be differentiated based on the chest X-ray, the radiologist concluded the chest X-ray could also be consistent with the catheter taking a dive into the hemiazygos. A formal ultrasound study was then recommended and completed verifying the placement of the catheter in the left internal jugular vein (Figure 3). Given mispositioning and confirmation of placement in the venous system, the catheter was then removed at bedside with the patient in the Trendelenburg position without any complications. 

## Discussion

It is known that central catheter placement via the left internal jugular vein is at higher risk for mispositioning given the longer and more oblique course to the SVC. The left internal jugular must join the subclavian to become the brachiocephalic vein which then crosses the chest midline to drain into the SVC [4]. Understanding this anatomy allows for the immediate recognition of mispositioning on routine post-procedure chest X-rays, as demonstrated in this case. During the procedure, ultrasound was used to confirm the positioning of the catheter in the internal jugular but it was not useful in the visualization of the catheter as it enters deeper into the chest [5].

The ascending hemiazygos vein can be connected to the left brachiocephalic vein along the subclavian and internal jugular veins [6]. Here we see a very uncommon complication where the patient’s anatomical variant, a large hemiazygos, led to the mispositioning of the central catheter. Given the ability to visualize the catheter in the internal jugular during placement, another explanation for why the catheter did not cross the midline, which can also be seen with catheter placement into the carotid artery, was sought out. It is important to review past chest imaging, especially CT chest if available, to note any anatomical variations a patient may have that can contribute to catheter mispositioning when taking an internal jugular approach. Ultrasound can be used to confirm catheter placement in the venous system, especially with the thicker temporary dialysis catheters that can be seen on ultrasound [7]. Lastly, mispositioning in the venous system would warrant immediate removal and observation for complications such as bleeding [8]. Given the rarity of mispositioning in the hemiazygos, we found only one case report documenting removal under CT guidance [9]. CT angiography would have been able to locate the entirety of the catheter and the exact location of placement [10]. Using ultrasound for visual confirmation of the catheter in the venous system and then immediate removal after recognition of the mispositioning resulted in good results with no complications or significant bleeding from our patient. If mispositioning occurred in the arterial system, or venous placement was not able to be confirmed, this would have been a case that required immediate surgical consultation for catheter removal [11].

## Conclusions

In this case, familiarity with the anatomy of the left internal jugular helped in the immediate recognition of mispositioning on routine post-procedure chest X-rays. Placing the central line with ultrasound increased confidence that the line was indeed in the left internal jugular vein even though the chest X-ray appeared consistent with arterial placement. A review of the patient's chest imaging for anatomical variants led to the discovery of a large hemiazygos vein. Mispositioning of a central line is more common with entering the left internal jugular vein compared to other vein sites and ultrasound is an important tool to confirm placement in the venous system. Ultrasound is also an important tool for surveying the possible veins for central catheter placement. Selecting the most optimal vein for catheter placement depends on vein anatomy, depth and diameter, and consideration of the more common complications associated with each possible catheter site. It is important for a provider to be familiar with both the anatomy and possible complications associated with central line placement when caring for patients with central venous access.
